# Assessment of Potential Factors Influencing Attention-Deficit/Hyperactivity Disorder Drug Adherence: A Database Study

**DOI:** 10.3390/ijerph22050716

**Published:** 2025-05-01

**Authors:** Ilse Truter, Judith Regnart, Anneke Meyer

**Affiliations:** Drug Utilization Research Unit (DURU), Department of Pharmacy, Faculty of Health Sciences, Nelson Mandela University, P.O. Box 77000, Gqeberha 6031, South Africa; judith.oettle@gmail.com (J.R.); annekemeyer4@gmail.com (A.M.)

**Keywords:** attention-deficit/hyperactivity disorder (ADHD), methylphenidate, antidepressant, mood disorder, adherence, proportion of days covered (PDC)

## Abstract

First-line treatment for Attention-Deficit/Hyperactivity Disorder (ADHD) is pharmacological but is associated with poor success rates in adults. The potential to discontinuously use stimulants may confound adherence assessment. Approximately 30–50% of adults with ADHD will experience depressive episodes, and associated treatment with antidepressants is generally recommended. It can therefore be expected that patients with a formal F90 diagnosis would show higher medication adherence than patients without a diagnosis and that the simultaneous use of antidepressants would increase adherence to ADHD medication. The primary aim was to explore the influence of factors of ADHD diagnosis and comorbid antidepressant use on stimulant adherence. A retrospective, longitudinal pharmacoepidemiological study was conducted on South African community pharmacy dispensing records for 2012–2016 for all patients aged between 18 and 40 years with any record of receiving a drug classified as “Central nervous system other” by the MIMS in 2015. Patients endorsed with an ADHD-linked diagnostic code (F90) were identified and contrasted with those receiving ADHD-indicated medication in the absence of a confirmatory diagnostic code. Two methods were applied to assess adherence to ADHD and/or depression treatment drugs: monthly medicine plotting and Proportion of Days Covered (PDC). Patients were classified as being more or less adherent based on monthly medicine plotting criteria. A study population of 89 patients was identified: 50 had F90 diagnostic codes and 39 were classified as “Non F90”. Adherence as measured based on PDC was generally higher for antidepressant use than for methylphenidate for patients classified as being more adherent. A trend towards higher consumption of antidepressants was shown for the treatment-adherent group. Diagnostic code distinction revealed significantly higher adherence rates to methylphenidate for F90 code patients. Adherence rates to antidepressants appeared to be generally higher for non-F90 patients. Many factors may influence adherence to ADHD-indicated drugs; however, the impact of a confirmed diagnosis may be a strong determinant of motivation to be adherent to ADHD pharmacotherapy.

## 1. Introduction

Attention-Deficit/Hyperactivity Disorder (ADHD) is a neurodevelopmental condition that presents in childhood and can persist into adulthood [1]. Adult ADHD contrasts from the typical childhood presentation; impulsiveness- and inattention-related symptoms often remain, but hyperactive behaviours often diminish, with them being replaced by inner restlessness [1,2].

Adult ADHD has been incorporated into the *Diagnostic and Statistical Manual of Mental Disorders, fifth edition* (*DSM-5*) [1]. ADHD is, however, terminology limited to the DSM, with the alternative notable diagnostic criteria source, the World Health Organization’s (WHO) International Classification of Diseases (ICD), referencing Hyperkinetic Disorder in its tenth edition (ICD-10) [1,2,3]. ADHD has replaced Hyperkinetic Disorder in ICD-11 [4]. For the purposes of this study, “F90” will be used. F90 refers to ADHD as classified under ICD-10.

First-line treatment is pharmacological [5,6]. Methylphenidate, a central nervous system stimulant, and atomoxetine, a selective noradrenaline reuptake inhibitor, are the two drugs indicated for ADHD management in South Africa [2,5,7,8]. The rate of success of pharmacological treatment in adults is often poor, however; approximately 50% of patients fail to adhere to or prematurely discontinue therapy [5,6].

This may be due to drug treatment being effective for core ADHD symptoms but not against functional symptoms of time management or planning; thus, treatment may be considered ineffective against desired outcomes, leading to discontinuation [6]. Gajria and colleagues’ [5] systematic review could not definitively determine the rationale behind adherence challenges in adult ADHD; however, it is suggested that drug tolerability has a greater influence over poor adherence than factors of social stigma or drug efficacy.

A substantial body of empirical research highlights the complex psychological, cognitive, and interpersonal determinants of medication adherence, which constitutes a major public health concern across a range of chronic conditions. Depression has been consistently shown to impair adherence, while the presence of robust social support can serve as a protective factor [9]. Patient beliefs about medication also play a central role; individuals tend to balance the perceived necessity of a treatment against concerns related to potential adverse effects or long-term harm [10,11]. Communication between patients and healthcare providers further influences outcomes, with effective physician communication linked to a 19% improvement in adherence [12]. At the behavioural level, adherence is hindered by a range of factors including emotional burden, forgetfulness, perceived stigma, and disruption of daily routines [13]. Psychological and cognitive mechanisms are also critical; emotional distress, cognitive dissonance, and decision fatigue can significantly undermine treatment engagement [14]. Moreover, adherence is closely associated with how individuals perceive and mentally represent their illness, including their beliefs about causality, control, and consequences [15]. Finally, adherence behaviour is shaped by motivational and volitional factors—patients are more likely to adhere when they feel a sense of agency, understanding, and intentionality regarding their treatment regimen [16]. Collectively, these findings underscore the need for multifaceted, psychologically informed interventions that address both structural and perceptual barriers to adherence within diverse patient populations.

In previous studies, investigators have given consideration to comorbid psychiatric disorders and the option to discontinue use of stimulant drugs to potentially impact pharmacological adherence assessment and its interpretation [5,6]. Approximately 30% to 50% of adults with ADHD will experience depressive episodes [17,18], with associated pharmacological treatment not being interrupted due to the potential risk of abrupt withdrawal precipitating antidepressant discontinuation syndrome [19]; it is contemplated that concurrent mood disorder pharmacotherapy with antidepressants may influence ADHD-indicated drug adherence.

Possible discontinuous stimulant drug use allows for observation of “drug holidays”, structured treatment interruptions for defined periods, which should not be interpreted as non-adherence [20]. Guidelines for applying stimulant drug holidays in ADHD are specific for children and adolescents [21]. Data relating to drug holidays in adult ADHD subjects are limited; however, a study by Polyzoi and colleagues [22] indicated that drug holidays observed in their study population were mostly unintended breaks.

Adherence is the extent to which a patient appropriately follows medication use instructions as prescribed with respect to administration frequency, dosage timing, and strength [23,24]. Commonly applied metrics for adherence measurement allow for the calculation of medication possession or gaps in medication availability [25]. Proportion of Days Covered (PDC) is a popularly applied possession-based adherence measure that is easily calculated and provides an estimation of the proportion of days’ supply obtained over a predetermined observation period [25]. Considering poor adult ADHD treatment adherence, the possible influence of comorbid psychiatric conditions with associated pharmacological treatments, and the potential to observe stimulant treatment holidays, ADHD-indicated medication adherence patterns in adults present an emerging field of interest. With this background, it was expected that patients with a formal F90 diagnosis would show higher medication adherence than patients without a diagnosis and that the simultaneous use of antidepressants would increase adherence to ADHD medication.

The primary aim of this investigation was therefore to explore adherence to stimulant therapy through analysis of a medical aid administration database.

The specific objectives were as follows:

- Identify patients with confirmed ADHD diagnoses versus those prescribed stimulants under other diagnostic codes;
- Compare whether differences in medicine adherence are noted between diagnostic groups;
- Compare whether adherence to methylphenidate therapy is modified by antidepressant use.

## 2. Materials and Methods

South Africa has a public (government or state) and private healthcare sector. The private health care sector covers approximately 17% of the South African population. This study was conducted on a section of the private healthcare sector using an electronic database. The database consists of between 4 to 5 million records for medicines per annum. Assumptions of normality and homoscedasticity were met, validating the use of parametric tests.

A retrospective, longitudinal study design was achieved with analysis of the South African Managed Care Group claims database, representing claims against medical aid schemes managed by the group. Specific data were requested for all members of the schemes aged between 18 and 40 years with any record of having had a drug classified as “1.1.3 Central nervous system other” according to the Monthly Index of Medical Specialities (MIMS) [26] classification system dispensed for them between January and December 2015. Drugs in this classification were methylphenidate and atomoxetine, both indicated for ADHD treatment, and flumazenil, used to reverse the effects of benzodiazepines. This was a longitudinal study. Patients were identified in 2015 (index year) and then followed over five years (from 2012 to 2016).

For the purposes of this study, patients endorsed with an ADHD-linked diagnostic code (F90) were identified and contrasted with those receiving ADHD-indicated medication in the absence of a confirmatory diagnostic code (“F90” refers to ADHD as classified under ICD-10).

A total of 120 patients were initially identified upon receipt of the database, with subsequent removal of the records of eight subjects who were ineligible for inclusion as a result of being 17 years of age in 2012. A further 23 patients were identified as only having had flumazenil dispensed of the three drugs included in the applied drug classification, thus not having had any ADHD-indicated drug dispensed to them or any confirmation of the presence of ADHD as per applied ICD-10 codes.

**Figure 1:**
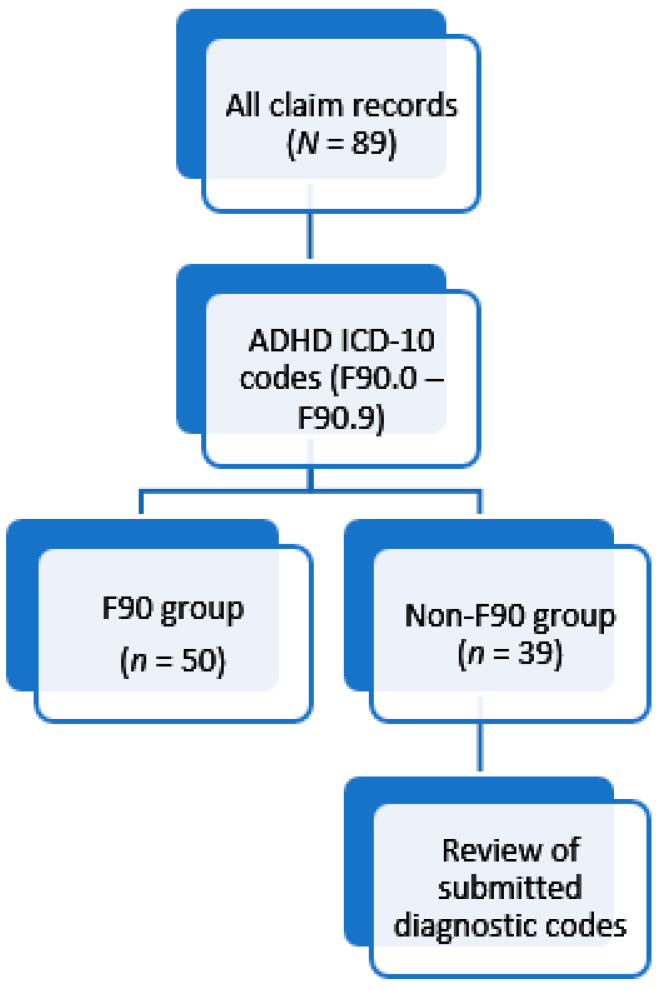
Identification of the ADHD-diagnosed group against subjects who received ADHD-indicated drugs without any history of a confirmed ADHD diagnosis over the study period.

A study population of 89 subjects was thus identified. Identification of individuals with a confirmed ADHD diagnosis was achieved with review of WHO ICD-10 codes submitted against the claims, as summarised in Figure 1.

Two methods were applied to assess adherence in the identified patient groups.

(i) Monthly medicine plotting

Given the different recommendations regarding continuous versus discontinuous use between stimulants and antidepressants, all drug issuance records for methylphenidate, atomoxetine, and antidepressants were plotted to indicate the month of issuance and total units of each drug dispensed per month. This was checked against actual dispensing dates, with consideration given to total days between dispensing dates and quantity of units dispensed to determine the approximate daily dose per drug and number of days coverage represented in order to consolidate the issuance records into an overview of monthly use and allow for analysis of usage frequency. Rules for assessing if claims performed in a single month could be attributed to the immediately preceding or proceeding calendar month for the purposes of plotting the usage pattern were applied as detailed in Table 1 (no changes were made to the actual dispensing records).

In instances where the number of units dispensed could not be empirically linked to a standard dosage, a default of one unit per day was assumed unless evidence to support a logical alternative was available. Any issues where the quantity supplied indicated short-term use were noted against the individual patients to prevent interpretation of short supplies representing a month’s medicine coverage. Tricyclic antidepressant usage identified was often misaligned with application to treat depression, as the dosages and durations were for short-term usage, lower than the accepted antidepressant dosage, or linked to an alternative ICD-10 code. Thus, such issuances were excluded from analysis.

Upon completing usage pattern plotting, the total number of months during which medication had been supplied was calculated for each subject for all investigated medications. A group of patients was identified as receiving medicines more consistently by applying these criteria:

- Receiving 12 or more monthly issues of either drug type over the five-year period.
- Receiving a minimum of seven monthly issues within 12 months.

This group of patients will be referred to as TAG—“treatment adherent group”.

(ii) Proportion of days covered

PDC describes the comparative portion of days during which a patient has a supply of a drug available within a study interval [24]. PDC is considered more accurate for adherence assessment when a person is prescribed multiple drugs within a class [24]; thus, its application to the identified groups allows for further exploration of the trends identified based on monthly plotting.

Overview of adherence patterns from monthly plotting showed that the drug(s) and assumed dosages could be varied, and it was not possible to discern the recommended therapy duration as required by individual prescribers. Thus, standardisation of a 90-day period after issuance of either drug type was applied, with calculation of average PDC for each patient per drug therapy type. The possibility for PDC to be unduly elevated is noted; however, the application of calculating the average PDC for patient groups is considered to minimise the impact of such outliers on the overall analysis. Rules were applied to guide the PDC assessment period durations to better represent the observed prescription filling trends to consistently determine the appropriate calculation in a manner to reduce over- or underestimation of PDC as a result of fluctuating regular or inconsistent usage. These rules are indicated in Table 2.

For purposes of comparison with methylphenidate’s Defined Daily Dose (DDD), the average Prescribed Daily Dose was calculated for each subject as a function of the number of days of drug cover.

Ethical approval to conduct this study was obtained from the Research Ethics Committee (Human) of Nelson Mandela University (H14-HEA-PHA-081) on 11 December 2014. De-identified records were obtained in Microsoft Excel^®^ and analysed.

## 3. Results

Demographic representation of the sample population is summarized in Table 3. Both groups are almost evenly representative of both genders. The F90 group represents more subjects overall, and for each year, these subjects are, on average, slightly younger than the non-F90 group. The F90 group displays greater variation in the average ages for each gender within a group.

The composition of the TAG versus the more irregular issuance in the remaining patients, with consideration of the ICD-10 diagnostic code-based division of patients, is shown in Table 4. Distinction is made between patients receiving only methylphenidate and those receiving both methylphenidate and antidepressants over the five-year period. Atomoxetine use was limited to one patient for a short duration of therapy; thus, these data were not incorporated into the analysis. The F90 group comprises the larger portion of the TAG (75.00%, *n* = 32), with use of both methylphenidate and one or more antidepressants being more frequent than methylphenidate monotherapy for both the F90 and non-F90 patients within the TAG (66.67%, *n* = 24 and 87.50%, *n* = 8). Adherence, as measured based on average PDC, was higher for antidepressant therapy than methylphenidate for both the F90 and non-F90 patients on both drug types within the TAG, as can be observed in Table 4. However, adherence to methylphenidate was on average 32.81% greater for the F90 group than the non-F90 group, while the non-F90 group showed 16.09% greater adherence to antidepressants than the F90 group. Regular usage of methylphenidate in the absence of an antidepressant was more readily observed for the F90 group (88.89%, *n* = 9), with adherence to methylphenidate monotherapy being 12.33% higher on average than when in combination with an antidepressant, and most of these subjects were male (66.67%, *n* = 9). Conversely, a greater proportion of the TAG F90 female subjects made use of both drug types than male subjects (68.75%, *n* = 16), with a 16.88% higher average antidepressant adherence and marginally higher methylphenidate adherence. The non-F90 portion of the TAG on both drug types comprised mostly male subjects (85.71%, *n* = 7), with a good average adherence for antidepressant therapy, which was 25.58% higher than for the F90 TAG male subjects, but adherence to methylphenidate was 22.58% lower. Consolidation of these comparisons between regular and irregular drug use revealed that most divisions within diagnostic code-defined groups tended more towards irregular use of medication, with the exception of female subjects in the F90 group, most of whom received both methylphenidate and antidepressants (84.62%, *n* = 13). Overall, receipt of methylphenidate without antidepressant medication was slightly more frequently observed (62.92%, *n* = 89).

To further investigate the usage patterns of the TAG and whether there was a greater tendency towards methylphenidate or antidepressant therapy, the total number of months representing methylphenidate therapy versus antidepressant therapy was determined. These were proportioned for each member individually in the TAG to indicate the drug type more regularly received, with average proportions calculated and reported alongside the associated average PDC. These results are shown in Table 5.

Overall, there was a trend towards higher consumption of antidepressants and greater adherence to antidepressant therapy. This was most notably skewed by the drug receipt trend of the non-F90 group, in which subjects were receiving approximately five months of antidepressant medication for every one month of methylphenidate on average. The monthly distribution between antidepressants and stimulants for male subjects was almost equal, with only a 3.13% difference in the amount received.

Comparisons across the entire study population without distinction related to interpreted adherence consistently showed that antidepressant adherence was greater than that observed for methylphenidate (refer to Table 6). Statistical analysis was performed using Fisher’s exact test to verify if the observed trends were statistically significant for defined groups within the study population. Adherence rates to antidepressants appeared to be generally greater for the non-F90 group; they were not statistically significant, however (*p* = 0.39) (*n* = 33). It was noted that antidepressant adherence for the F90 females was 2.88% higher than for their non-F90 counterparts. Closer evaluation of the trends for the F90 females (*n* = 12) indicated greater methylphenidate adherence when taken concurrently with antidepressants, with this result approaching significance (*p* = 0.08).

A logistic regression analysis was performed using the TAG as a binary dependent variable to test drug therapy adherence probability across the entire study population (*n* = 89). With application of the Type 3 Likelihood-ratio test, it was shown that treatment adherence was not significantly different for the two diagnostic groups (*p* = 0.1325). The presence of an ADHD diagnosis was shown to improve the odds of being treatment adherent by a factor of two, although this finding was also not statistically significant (*p* = 0.1417). Application of Fisher’s exact test to assess MPH-specific adherence, as measured based on MPH PDC, indicated that the greater adherence measure scores associated with the F90 patients were significant compared to non-F90 group adherence (*p* = 0.005) (*n* = 89). Adherence to drug therapy was found to be significantly improved by a factor of 20 with the presence of antidepressant therapy (*p* = 0.000022) *(n* = 89).

This investigation was expanded to identify independent variables associated with predicted improved adherence with the application of the Wald Chi-Square test. This revealed female gender, confirmed ADHD diagnosis, and concurrent antidepressant therapy as being predictive of improved adherence. Through stratification of the study population into four age groups based on reference year age (20–24 years, 25–29 years, 30–34 years and 35–39 years) and application of the youngest group as the base level age, it was shown that improved adherence could be predicted at the 5% level of significance for the age group of 30–34 years.

The PDD for methylphenidate was not significantly different for the different diagnostic groups with application of Fisher’s exact test (*p* = 0.12) (*n* = 89); however, it was generally higher for the F90 group. PDD measurements were combined without distinction for the presence or absence of concurrent antidepressant therapy, and these results are indicated in Table 7. Overall, methylphenidate PDD for the entire study population was approximately 30 mg (*N* = 89). The noted higher average PDDs for the F90 group were repeated, with the average dosage being 13.22% higher for the F90 subjects (*n* = 50).

## 4. Discussion

This study explored the influence of factors of ADHD diagnosis and comorbid antidepressant use on stimulant adherence. However, it is acknowledged that there may be other potential confounding factors, such as socioeconomic status, healthcare access, and variations in prescribing practices, that could influence adherence rates. These factors were not investigated in this study since an electronic database was used, in which these factors were not available.

Overall, antidepressant adherence rates appeared to be generally higher than those of methylphenidate, and the presence of antidepressant therapy was found to be associated with significantly improved adherence to drug therapy. This finding was interesting in light of the presence of psychological illness, with emphasis on depression, being considered a major predictor of poor adherence [14]. However, the presence of cognitive impairment, which can be evident in both ADHD and mood disorders [1], is also associated with poor adherence [14]. These factors could aid explanation of the observed adherence rates being poor when considered against the convention of 80% adherence and higher being acceptable [14]. Nonetheless, the rates observed in this investigation for antidepressant adherence were comparable to the mean adherence rate of 65% reported in the systematic review by Cramer and Rosenheck [27].

A confirmed ADHD diagnosis was associated with improved adherence to methylphenidate therapy, as measured based on PDC, with a predicted improvement in drug therapy adherence by a factor of two, whether alone or in combination with an antidepressant. This could be related to the acceptance of the diagnosis of ADHD, with understanding of its treatment as a necessity. Kleppe and colleagues [28] reported that necessity associations were correlated with the greatest mean adherence rates to therapy in their investigation of subjects taking chronic medications for diabetes, cardiovascular diseases, and hypertension. Emilsson and colleagues [29] assessed the influence of beliefs about medicine and ADHD perception together with objective factors in adolescents on long-term ADHD prescription medication and found that a positive differential of beliefs in the necessity against the concerns of medication, together with experiencing fewer side effects, was significantly associated with greater adherence. This study also provided evidence that females tended to report stronger belief in treatment necessity than males, associated with greater adherence [29].

The overall adherence to antidepressant therapy in the non-F90 group was 7.5% higher than in the F90 group, coupled with a 30.8% lower methylphenidate adherence rate for concurrent antidepressant therapy and a 28.1% lower adherence rate for methylphenidate monotherapy. This could also indicate the influence of necessity association on adherence, given that the intention for using methylphenidate was not apparent from the available diagnostic codes. It is noted that methylphenidate usage in this group could be for the purpose of treating ADHD symptoms or as monotherapy or an augmenting agent for depression [30]. There is also the potential for these adherence patterns to be related to a habitual view of medicine taking, which could be associated with recommended continuous dosing of antidepressants, with the literature indicating that adherence is improved when the activity of taking medicine does not require cognitive thought [28].

The higher rates of antidepressant adherence over methylphenidate in both diagnostic groups, together with the proportion of treatment months in the TAG being mostly skewed towards antidepressants and the significantly improved odds of being adherent to drug therapy in the presence of treatment with antidepressant supports the notion that a combination of necessity belief and habitual medicine use could explain these patterns. Consideration is given to the findings reported by Safren and colleagues [31] wherein higher adherence rates were negatively correlated with ADHD symptom severity, indicating impairment of adherence with aggravated ADHD symptomology. However, the improved adherence to antidepressant therapy in this study indicates that higher adherence rates are possible despite the inherent risk to adherence posed by ADHD presence. It therefore seems likely that poor methylphenidate adherence may result from factors over and above ADHD symptoms and their severity.

As suggested by Semerci [6] and Gajria and colleagues [5], lower rates of methylphenidate adherence could be attributable to low medication satisfaction derived from insufficient efficacy for functional symptoms or poor tolerability. Due consideration is also given to potential confounding on true methylphenidate adherence determination by drug holiday observance [5]. The former factors are echoed in two of the 12 major predictors of non-adherence summarised by Osterberg and Blaschke [14]: medication side effects and a patient’s lack of belief in treatment benefit. However, other factors listed by Osterberg and Blaschke [14] include barriers to care or medications, missed appointments, and medication cost, and these may be overlooked or under-reported in the ADHD treatment setting when great emphasis is placed on drug tolerability and treatment stigma [5]. Within South Africa, legislation allows for prescriptions for antidepressants (schedule 5) to be repeated for a six-month duration, whereas methylphenidate is a schedule 6 drug requiring new prescriptions every month [32]. Consideration is given to the greater level of coordination and planning, and the potential for incurring additional costs associated with this distinction between the medicine types and the possibility for these factors to compound challenges for methylphenidate adherence.

With the culmination of all of these factors, it is considered that it was hypothesized that concurrent antidepressant therapy could improve methylphenidate treatment adherence; however, it was identified that the presence of antidepressant therapy in itself was associated with significantly improved adherence to pharmacotherapy for the studied population. Given the various challenges associated with methylphenidate therapy, it is suggested that when poor methylphenidate adherence (with associated poor therapeutic outcomes) is identified in patients, off-label treatment of ADHD with antidepressant therapy may be associated with improved treatment adherence. This could be applied with appropriate agents as augmenting therapy in combination with methylphenidate or application as monotherapy [33].

The findings of this study should be considered against the following limitations:

1. The claims included in the dataset were only representative of those submitted to the included medical aids’ administration for consideration. It is possible that subjects may have paid privately for their medication, thus possibly confounding adherence measurements.
2. ICD codes were not available for 2012 and few were indicated for 2013. Thus, identification of the F90 group may have omitted members who were assigned to the Non-F90 group based on the lack of an appropriate diagnostic code.
3. The field indicating the number of days supply was only available for claims in 2016. These were not applied in the analysis for the purpose of consistency in assessment method and due to noted inaccuracies in the supply duration provided.
4. Dosage instructions for the medicines were not available.
5. Consumption of medication was based on dispensing records; it was not possible to determine if medication was taken as prescribed.
6. There was no control for psychological comorbidities other than depression, experience with medication, or treatment preferences.
7. Lastly, the small sample size impacted on generalizability and margins of error.
8. Other potential confounding factors, such as socioeconomic status, healthcare access, and variations in prescribing practices that could influence adherence rates, were not considered in this study.

It is recommended that more work be carried out in this field. A recent study by Hosseinnia et al. [34] reported on applications (apps) for the management of ADHD. The authors conducted a systematic review and concluded that apps can potentially be adjunctive instruments for treating ADHD. Apps can potentially become a valuable tool in increasing drug adherence in ADHD.

## 5. Conclusions

With consideration of the noted limitations, it can be deduced that many factors can influence adherence to ADHD-indicated drugs and drug therapy in general. With specific reference to ADHD-indicated pharmacotherapy, the impact of a confirmed diagnosis may be an important determinant of motivation to be adherent; however, general pharmacotherapeutic adherence in the presence of ADHD may drastically improve with the application of antidepressant agents. Emphasis is therefore placed on the need to follow the appropriate diagnostic procedure for ADHD, with understanding of the condition, its implications, and the nature of the symptoms with reference to prescribing pharmacological management to align drug use with desired outcomes and consideration for interventive strategies in the event of poor pharmacotherapeutic adherence.

## Figures and Tables

**Table 1:** Rules applied for the determination of dispensing month reassignment when two dispensing dates were within a single calendar month.

Rule Number	Explanation of Rule
Rule 1	The number of days between antidepressant/ADHD medication issues was equal to 21 or more days, and either the preceding or proceeding month did not contain records of an issue misaligned with an otherwise stable pattern of usage for the same drug or drug class, strength, and quantity dispensed.
Rule 2	A regular trend for drug and dosage was observed with minimal outliers, and the total days between dispensing points were calculated against the total units dispensed and compared to determine if these matched the approximate daily dosage as per the regular trend observed.

**Table 2:** Rules applied to guide the duration of PDC assessment periods.

Assessment Period	Circumstance	Example
90 days	Standard applied duration, unless a requirement to adjust upwards or downwards was indicated by actual dispensing dates.	Not applicable.
Over 90 days	The end date of the total number of days covered for two or three consecutive issues of medicine exceeded the 90-day cut-off and/or the number of days between the end date and the date of the next issue of medicine exceeded 90 days.	First issue: 16 January 2014 Second issue: 18 February 2014 Third issue: 20 March 2014 Fourth issue: 23 April 2014 Total: 97 days between the first issue and the date of THE fourth issue. The date of THE fourth issue calculated from THE third issue would be 19 April 2014.
Up to 119 days	Medicine noted as having been dispensed shortly before the 90-day cut-off as a result of delays between consecutive issues which exceeded the number of days which were covered, and/or no further issue of medicine was carried out in the preceding month, and the calculated end date of the final issue fell within this range.	First issue: 3 August 2014 Next issue: 30 October 2014 Calculated proceeding supply date: 29 November 2014 Total: 118 days
120–129 days	The last issue of medicine falls appropriately within the approximate 90-day period, but the next issue date of the medicine, which would indicate the assessment end date, differs by over 119 days but less than 130 days, and the usage pattern is regular.	First issue: 28 Apr 2015 Second issue: 3 June 2015 Third issue: 3 July 2015 Fourth issue: 31 August 2015 Total: 125 days; the next supply date after 3 July 2015 would be 2 August 2015, representing a total of 96 days, but the actual date for consecutive issuing of the same medicine was slightly later.
Less than 90 days	If one month within an assessment period of 90 days had no issue, however the number of days covered within the next assessment period was equal to or greater than 60, with its first issue date being slightly before the end of the first assessment period (approximately 90 days), the assessment period was closed as of the date of the first issue of medicine within the next assessment period and could represent less than 90 days.	First issue: 25 February 2013 Second issue: 13 April 2013 Third issue: 18 May 2013 Fourth issue: 27 June 2013 Fifth issue: 17 August 2014 Total: 82 days between the first issue and the third issue. The next assessment period has 60 days of coverage over a 91-day period.
End date for database records	Medicine issues at the end of 2016 were calculated against the number of days that would have passed up until 1 Jan 2017, as the absence of a proceeding issue date leaves the assessment for the days covered indeterminate.	First issue: 2 November 2016 Second issue: 5 December 2016 End date: 1 January 2017 Total: 60 days, with confirmed coverage of 57 days within the period.

**Table 3:** Study Population Demographic Representations. Referenced Age Is In Years In All Instances.

Patient Group	2012	2013	2014	2015	2016	Total
F90 (*n*)	34	38	41	50	45	50
Age range	18.24–36.51	19.24–37.51	20.24–38.51	20.63–39.51	21.63–39.56	-
Average age	25.45 ± 6.13	26.6 ± 6.10	27.57 ± 5.94	28.19 ± 5.68	29.01 ± 5.63	-
Female (*n*)	20	20	22	23	21	23
Age range	18.24–36.51	19.24–37.51	20.24–38.51	20.63–39.51	21.63–39.56	-
Average age	25.74 ± 6.23	26.74 ± 6.07	27.3 ± 6.13	27.97 ± 6.2	28.59 ± 5.9	-
Male (*n*)	14	18	19	27	24	27
Age range	18.30–35.01	19.30–36.13	20.30–37.13	21.30–38.13	22.30–39.13	-
Average age	25.03 ± 6.19	26.44 ± 6.31	27.88 ± 5.87	28.38 ± 5.31	29.39 ± 5.47	-
Non–F90 (*n*)	23	29	32	39	36	39
Age range	18.57–35.26	19.57–36.26	20.57–37.26	21.57–39.47	22.57–40.47	-
Average age	27.17 ± 5.52	28.54 ± 5.39	29.01 ± 5.47	30.07 ± 5.41	30.84 ± 5.33	-
Female (*n*)	14	16	18	19	17	19
Age range	18.57–35.26	19.57–36.26	20.57–37.26	21.57–38.26	22.57–39.26	-
Average age	26.17 ± 5.95	26.74 ± 5.7	27.36 ± 5.6	28.32 ± 5.44	29 ± 5.29	-
Male (*n*)	9	13	14	20	19	20
Age range	19.47–35.17	20.47–36.17	21.47–37.17	22.32–39.47	23.32–40.47	-
Average age	28.72 ± 4.68	30.76 ± 4.19	31.12 ± 4.67	31.74 ± 4.94	32.48 ± 4.93	-

**Table 4:** PDC Measurements For Different Population Groups Defined By Adherence, Gender, Drug Therapy, And Diagnostic Code (*N* = 89). AD = antidepressants; F90 = ICD-10 code for ADHD diagnosis; MPH = methylphenidate; PDC = Proportion of Days Covered.

	MPH & AD								MPH Alone						Total			
	Regular Use				Irregular Use				Regular Use			Irregular Use			Regular Use		Irregular Use	
	*N*	%	PDC		*N*	%	PDC		*N*	%	PDC	*N*	%	PDC	%	PDC	%	PDC
			AD	MPH			AD	MPH										
F90 (*n* = 50)	16	32.00	0.73	0.64	4	8.00	0.58	0.57	8	16.00	0.73	22	44.00	0.48	48.00	0.69	52.00	0.50
Female (*n* = 50)	11	22.00	0.77	0.65	1	2.00	0.31	0.67	2	4.00	0.73	9	18.00	0.49	26.00	0.71	20.00	0.49
% of females in group (*n* = 23)	-	47.83	-	-	-	4.35	-	-	-	8.70	-	-	39.13	-	56.52	-	43.48	-
% of females in group on antidepressants (*n* = 12)	-	91.67	-	-	-	8.33	-	-	-	-	-	-	–	-	-	-	-	-
Male (*n* = 50)	5	10.00	0.64	0.62	3	6.00	0.67	0.54	6	12.00	0.73	13	26.00	0.47	22.00	0.67	32.00	0.51
% of males in group (*n* = 27)	-	18.52	-	-	-	11.11	-	-	-	22.22	-	-	48.15	-	40.74	-	59.26	-
% of males in group on antidepressants (*n* = 8)	-	62.50	-	-	-	37.50	-	-	-	-	-	-	–	-	-	-	-	-
Non–F90 (*n* = 39)	7	17.95	0.87	0.43	6	15.38	0.64	0.44	1	2.56	0.71	25	64.10	0.38	20.51	0.65	79.48	0.43
Female (*n* = 39)	1	2.56	0.92	0.11	3	7.69	0.64	0.33	1	2.56	0.71	14	35.90	0.38	5.12	0.58	43.59	0.41
% of females in group (*n* = 19)	-	5.26	-	-	-	15.79	-	-	-	5.26	–	–	73.68	–	10.53	-	89.47	-
% of females in group on antidepressants (*n* = 4)	-	25.00	-	-	-	75.00	-	-	-	-	-	-	-	-	-	-	-	-
Male (*n* = 39)	6	15.38	0.86	0.48	3	7.69	0.64	0.55	0	0.00	-	11	28.21	0.38	15.38	0.67	35.90	0.45
% of males in group (*n* = 20)	-	30.00	-	-	-	15.00	-	-	-	-	-	-	55.00	-	45.00	-	55.00	-
% of males in group on antidepressants (*n* = 9)	-	66.67	-	-	-	33.33	-	-	-	-	-	-	-	-	-	-	-	-

**Table 5:** Proportions of drug types dispensed for the treatment-adherent group (*n* = 23).

	Antidepressant Portion	PDC	Methylphenidate Portion	PDC
All regular use (*N* = 23)	62.44%	0.77	37.56%	0.58
Female (*n* = 12)	62.79%	0.78	37.21%	0.60
Male (*n* = 11)	67.23%	0.76	32.77%	0.54
F90 regular (*n* = 16)	56.72%	0.73	43.28%	0.64
Female (*n* = 11)	59.70%	0.77	40.30%	0.65
Male (*n* = 5)	50.17%	0.64	49.83%	0.62
Non-F90 regular (*n* = 7)	83.64%	0.87	16.36%	0.43
Female (*n* = 1)	96.77%	0.92	3.23%	0.11
Male (*n* = 6)	81.45%	0.86	18.55%	0.48

**Table 6:** Comparison of average PDCs and methylphenidate average PDD for different treatment-, ICD-10 code-, and gender-defined groups in the study population (*N* = 89).

	Concurrent AD			MPH Monotherapy		
Entire study population (*N* = 89)	Overall	Male	Female	Overall	Male	Female
	*n* = 56	*n* = 30	*n* = 26	*n* = 33	*n* = 16	*n* = 17
Antidepressant PDC	72.3%	72.3%	72.4%	N/A	N/A	N/A
Methylphenidate PDC	55.0%	54.5%	55.6%	47.4%	48.8%	45.9%
MPH PDD (mg)	31.29	26.44	36.94	29.76	31.88	27.30
F90 population (*n* = 50)	Overall	Male	Female	Overall	Male	Female
	*n* = 20	*n* = 8	*n* = 12	*n* = 30	*n* = 19	*n* = 11
Antidepressant PDC	70.6%	67.1%	72.9%	N/A	N/A	N/A
Methylphenidate PDC	62.6%	59.2%	64.9%	54.5%	55.3%	53.3%
MPH PDD (mg)	32.09	26.34	37.32	30.53	32.28	27.64
Non–F90 population (*n* = 39)	Overall	Male	Female	Overall	Male	Female
	*n* = 13	*n* = 9	*n* = 4	*n* = 26	*n* = 11	*n* = 15
Antidepressant PDC	76.3%	78.8%	70.8%	N/A	N/A	N/A
Methylphenidate PDC	43.4%	50.3%	27.8%	39.2%	37.5%	40.4%
MPH PDD (mg)	26.50	26.75	24.10	27.39	29.06	26.75

**Table 7:** Overall MPH PDC and PDD for each diagnostic-code defined group with stratification for gender (*N* = 89).

	*n*	MPH PDC	PDD	N	MPH PDC	PDD	*n*	MPH PDC	PDD
		Overall	(mg)		Overall	(mg)		Overall	(mg)
		Entire Study Population			F90 Population			Non-F90 Population	
Overall	89	50.24%	30.42	50	57.77%	31.25	39	40.58%	27.12
Female	42	49.57%	31.46	23	59.33%	32.91	19	37.76%	26.61
Male	47	50.83%	29.53	27	56.44%	29.92	20	43.26%	27.69

## Data Availability

The dataset presented in this article is not available due to the ethical agreement signed by the university. Requests to access the dataset should be directed to the chairperson of the university’s research ethics committee.

## Abbreviations

The following abbreviations are used in this manuscript:

ADHD	Attention-Deficit/Hyperactivity Disorder
SUD	Substance Use Disorder
